# Characterization and Scaled-Up Production of Azido-Functionalized Silk Fiber Produced by Transgenic Silkworms with an Expanded Genetic Code

**DOI:** 10.3390/ijms20030616

**Published:** 2019-01-31

**Authors:** Hidetoshi Teramoto, Masatoshi Iga, Hiromi Tsuboi, Kenichi Nakajima

**Affiliations:** Division of Biotechnology, Institute of Agrobiological Sciences, National Agriculture and Food Research Organization (NARO), Ibaraki 305-8518, Japan; masatoshiiga@affrc.go.jp (M.I.); tsuboih552@affrc.go.jp (H.T.); kenchi@affrc.go.jp (K.N.)

**Keywords:** azidophenylalanine, *Bombyx mori*, click chemistry, silk fibroin, synthetic amino acid

## Abstract

The creation of functional materials from renewable resources has attracted much interest. We previously reported on the genetic code expansion of the domesticated silkworm *Bombyx mori* to functionalize silk fiber with synthetic amino acids such as 4-azido-L-phenylalanine (AzPhe). The azido groups act as selective handles for biorthogonal chemical reactions. Here we report the characterization and scaled-up production of azido-functionalized silk fiber for textile, healthcare, and medical applications. To increase the productivity of azido-functionalized silk fiber, the original transgenic line was hybridized with a high silk-producing strain. The F_1_ hybrid produced circa 1.5 times more silk fibroin than the original transgenic line. The incorporation efficiency of AzPhe into silk fibroin was retained after hybridization. The tensile properties of the azido-functionalized silk fiber were equal to those of normal silk fiber. Scaled-up production of the azido-functionalized silk fiber was demonstrated by rearing circa 1000 transgenic silkworms. Differently-colored fluorescent silk fibers were successfully prepared by click chemistry reactions, demonstrating the utility of the azido-functionalized silk fiber for developing silk-based materials with desired functions.

## 1. Introduction

Materials development based on naturally derived renewable resources is of great interest because such sources are independent of fossil resources and have lower impact on the environment [1,2,3]. Silk fiber produced by the domesticated silkworm, *Bombyx mori*, is one such natural resource and has the advantageous properties of mechanical toughness, elasticity, biocompatibility, and biodegradability [4,5,6]. The development of novel technologies to utilize *B. mori* silk could thus lead to the creation of high-performance bio-based materials for textile, healthcare, and medical fields.

We have been developing a novel technology to incorporate synthetic amino acids with unnatural functional groups into silk fibroin [7,8,9,10], which is the major protein component of *B. mori* silk and is regarded as a heterodimer of fibroin heavy chain (FibH; ~390 kDa) and fibroin light chain (FibL; ~26 kDa). The technology for incorporating synthetic amino acids into proteins is referred to as genetic code expansion, and it could enhance the utility of proteins as renewable materials. In the case of silk fibroin, incorporation of synthetic amino acids bearing azido groups endows the fibers with novel functionalities. Azido groups can act as selective chemical handles for further modifications with desired functional molecules for specific applications.

**Figure 1:**
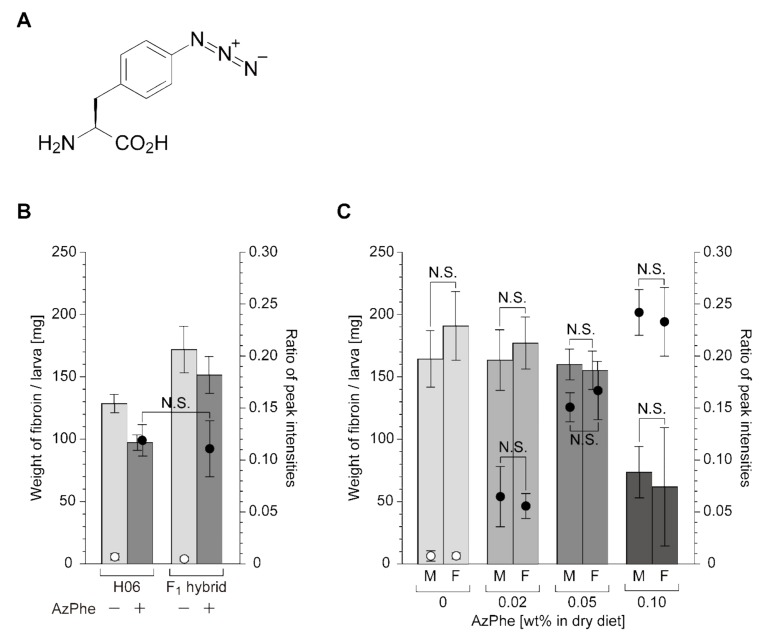
Fibroin production and the AzPhe incorporation rate. All values in this figure are the average of four independent experiments, where each experiment employed three male larvae. Bar plots represent the amounts of fibroin. Open and closed circles represent the ratio of peak intensities (870 Da/855 Da) in MALDI-TOF-MS analysis (Figure S3) of a FibL-derived peptide as an index of AzPhe incorporation [10]. The plots from the control experiments without AzPhe administration (open circles) show the levels of background signals. The error bars represent standard deviations (*n* = 4). N.S. stands for not statistically significant by *t*-test. (**A**) Chemical structure of 4-azido-L-phenylalanine, AzPhe. (**B**) Comparison between the H06 strain and its F_1_ hybrid with a high silk-producing strain, Nichi509 × Nichi510. Fifth instar larvae were fed a diet containing AzPhe (0 or 0.05 wt% in dry diet) from their third day until the start of spinning. Representative MALDI-TOF-MS spectra are shown in Figure S3. (**C**) Dependence on the amount of AzPhe in the diet. Fifth instar larvae were fed a diet containing different amounts of AzPhe (0, 0.02, 0.05, or 0.1 wt% in dry diet) from their third day until the start of spinning. Male (M) and female (F) larvae of the F_1_ hybrid were separately tested.

To achieve the in vivo incorporation of an azido-bearing synthetic amino acid, 4-azido-L-phenaylalanine (AzPhe) (Figure 1A), into silk fibroin, several mutants of *B. mori* phenylalanyl-tRNA synthetase (BmPheRS) which efficiently accommodate AzPhe as a substrate were selected in *E. coli* by a growth inhibition assay [10]. The selected mutants were expressed in the posterior silk glands (PSGs) of transgenic *B. mori* larvae. The PSGs are the organs where silk fibroin is synthesized. The transgenic line with the highest AzPhe incorporation efficiency was designated as the H06 line, which expresses the F432V mutant of BmPheRS [10]. When the H06 larvae were fed a diet containing AzPhe (0.05 wt% in dry diet) from the third day in their final (fifth) instar until the start of spinning, 6.6% of 35 phenylalanine (Phe) residues in silk fibroin, corresponding to an average 2.3 residues per molecule, were replaced to AzPhe.

The azido groups of AzPhe in silk fibroin were confirmed to be available for bioorthogonal modifications by the click chemistry reaction, azido-alkyne [3+2] cycloaddition [11,12]. The reaction proceeded in three different material forms: fiber, film, and porous sponge [9]. The azido groups can be converted to alkyne groups by reaction with a bifunctional compound, DBCO-PEG4-DBCO [13].

The H06 line was derived from a small-sized strain suited for transgenesis experiments and thus produced smaller cocoons and thinner fibers than strains used for practical silk production. In this study, the H06 line was hybridized with a high silk-producing strain to achieve scaled-up production of the azido-functionalized silk fiber using a conventional automatic reeling machine. The F_1_ hybrid produced circa 1.5 times larger amounts of silk fibroin, and the incorporation efficiency of AzPhe was maintained after hybridization. The mechanical properties were not affected by AzPhe incorporation. We demonstrated the preparation of differently-colored fluorescent silk fibers by click chemistry reactions.

This study realized a rare opportunity to apply genetic code expansion methodology to the production of proteins containing synthetic amino acids on an industrial scale.

## 2. Results and Discussion

### 2.1. Fibroin Production and AzPhe Incorporation

The H06 line was generated based on the MCS601 strain, which is a relatively small-sized strain appropriate for genetic transformation experiments. The cocoon shells of MCS601 are thinner than those of the high silk-producing strains generally used for silk production on an industrial scale. To improve the productivity of the azido-functionalized silk fiber, the H06 line was hybridized with a high silk-producing wildtype strain, Nichi509 × Nichi510 [14]. When a transgenic line bearing homozygous transgene(s) is hybridized with a wildtype strain, the copy number of transgenes decreases by half. In the case of the H06 line, it has one homozygous BmPheRS mutant (F432V) gene; that is, it has two copies of the transgene in its genome. Its F_1_ hybrid with a wildtype strain is heterozygous and has one copy of the transgene. A decrease in the copy number would lead to decreased expression of the mutant enzyme, which might lead to lowered incorporation of AzPhe into silk fibroin.

Male larvae of the H06 line and its F_1_ hybrid with Nichi509×Nichi510 were fed a diet containing AzPhe (0 or 0.05 wt% in dry diet) from their third day of fifth instar until the start of spinning. No adverse effects on their larval growth were observed (Figure S1); the F_1_ hybrid grew heavier than the H06 line as expected. Larger amounts of diet were required for maturation of the F_1_ hybrid than the H06 line (Figure S2). Figure 1B shows the fibroin production and AzPhe incorporation rate in the H06 and its F_1_ hybrid with Nichi509×Nichi510. As reported previously [10], the fibroin production decreased upon AzPhe administration. The F_1_ hybrid produced larger amounts of fibroin than the H06 line, whereas the AzPhe incorporation rate, estimated from the ratio of peak intensities in matrix-assisted laser desorption/ionization time-of-flight mass spectrometry (MALDI-TOF-MS) spectra (Figure S3), was not significantly different between the two lines.

It is noteworthy that the AzPhe incorporation rate was not affected by hybridization despite the decrease in the copy number of the transgene encoding the BmPheRS mutant (F432V). We speculated that the concentration of the mutant enzyme in PSG cells was high enough for sufficient enzymatic activity to aminoacylate tRNA^Phe^ with AzPhe even when the copy number of the transgene decreased by half.

In the above comparison, only male larvae were used for the experiments to avoid data variation due to sex differences. However, in industrial production of silk fiber, both male and female larvae were reared without discrepancy. Varied amounts of AzPhe (0, 0.02, 0.05, or 0.1 wt%) were then administered separately to male and female larvae of the F_1_ hybrid, H06 × (Nichi509 × Nichi510), to investigate the sex difference as well as to verify the optimal AzPhe concentration in diet. Fibroin production and the AzPhe incorporation rate exhibited no statistically significant differences between male and female larvae (Figure 1C), demonstrating that both male and female larvae of the F_1_ hybrid were available for industrial silk production. We verified that 0.05 wt% AzPhe in dry diet was optimal to balance fibroin production and AzPhe incorporation (Figure 1C). This result was consistent with the previous result obtained with the H06 line [10].

### 2.2. Cocoon Reeling and Mechanical Property

Fifth instar larvae of the F_1_ hybrid were reared on a normal or an AzPhe-containing diet and their cocoons were harvested. Raw silk fiber was reeled out from the cocoons using a conventional automatic reeling machine. Characterization of the raw silk fiber revealed no significant differences in the fiber length, fiber size, or degumming ratio (Table 1). On the other hand, the raw silk ratio became significantly lower when AzPhe was incorporated into silk fibroin. AzPhe administration decreased the fibroin production, as shown in Figure 1B, which would be one reason for the lower raw silk ratio. Another reason might be that AzPhe administration influenced the weight of the pupae. However, the relationship between AzPhe administration and the weight of pupae has not been investigated.

Mechanical properties of the raw silk fiber (Young’s modulus, maximum stress, and maximum strain) were compared (Table 2). None of the properties were significantly changed by AzPhe incorporation.

**Figure 2:**
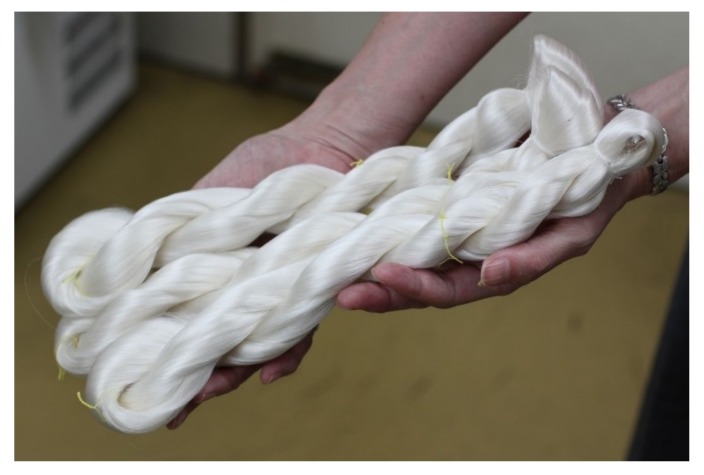
Picture of three bundles of the azido-functionalized raw silk fiber. Around 160 g of raw silk fiber was produced in total by rearing circa 1000 larvae of the F_1_ hybrid, H06 × (Nichi509 × Nichi510). The larvae were fed AzPhe-containing diet (0.05 wt% in dry diet) on their third day of fifth instar until the start of spinning.

In our system, AzPhe replaces a portion of the 35 Phe residues found in silk fibroin. Phe are not observed in the crystalline regions, which are mainly made of Gly-Ala repeats [15]. From the data in Figure 1C, the replacement ratio of Phe to AzPhe was calculated to be circa 6.8% in the F_1_ hybrid (including both male and female larvae) when 0.05 wt% AzPhe was added to the dry diet. The 6.8% replacement roughly corresponds to 2.4 residues of AzPhe in one silk fibroin molecule comprised of circa 5500 amino acids. We speculated that such a small number of AzPhe residues in the non-crystalline regions probably had a negligible impact on the mechanical properties of raw silk fiber. The azido-functionalized silk fiber produced by the F_1_ hybrid can thus be directly used for industrial applications via the same processing methods as normal raw silk fiber. As a trial to scale-up the production of the azido-functionalized silk fiber, we reared circa 1000 larvae of the F_1_ hybrid on an AzPhe-containing diet (0.05 wt% in dry diet) and obtained circa 160 g of raw silk fiber (Figure 2).

### 2.3. Click Modification

**Figure 3:**
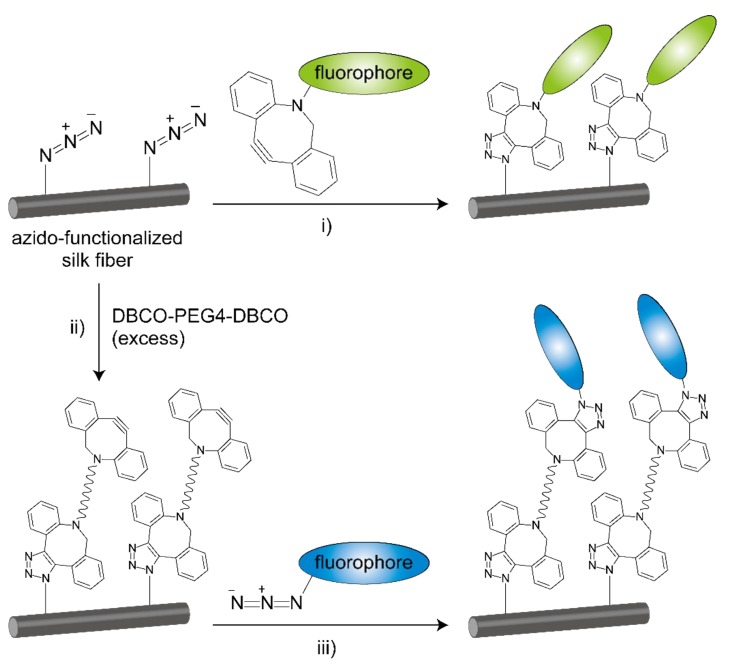
Modification of the azido-functionalized silk fiber with fluorophores by SPAAC. (i) SPAAC between azido groups in silk fibroin and DBCO groups in fluorescent reagents to form stable triazole rings. (ii) Azido groups in silk fibroin can be converted into DBCO groups by the reaction with excess DBCO-PEG4-DBCO. (iii) SPAAC between DBCO groups in silk fibroin and azido groups in fluorescent reagents.

The sericin coating of the raw silk fiber was removed by a degumming process to obtain pure fibroin fiber. To verify the reactivity of azido groups in fibroin, the degummed fiber was treated with fluorescent reagents bearing dibenzocyclooctyne (DBCO) groups. DBCO groups selectively react with azido groups by a click chemistry reaction, strain-promoted azido-alkyne cycloaddition (SPAAC) [11,12] (Figure 3).

**Figure 4:**
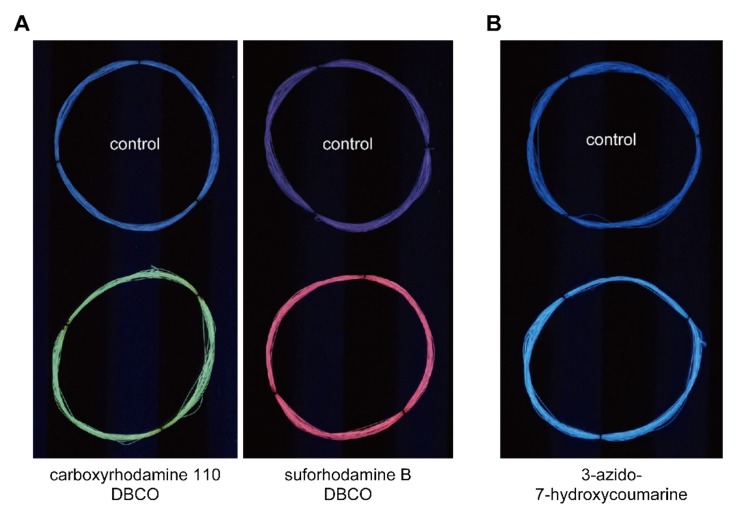
Preparation of differently-colored fluorescent silk fiber by modification of the azido-functionalized silk fiber with fluorescent molecules by SPAAC. The control fiber without AzPhe administration was subjected to the same reaction and washing processes. (**A**) Azido groups in silk fibroin were directly modified with DBCO-functionalized fluorescent reagents, carboxyrhodamine 110 DBCO (green) or sulforhodamine B DBCO (red). (**B**) Azido groups in silk fibroin were first converted into DBCO groups by the reaction with excess DBCO-PEG4-DBCO and then modified with an azido-functionalized fluorescent reagent, 3-azido-7-hydroxycoumarine (blue).

The azido-functionalized degummed silk fiber was soaked in a solvent containing green or red fluorescent reagent and reacted overnight at room temperature. The fiber was then thoroughly washed and dried. Distinct fluorescence was observed on the fiber, whereas the control fiber not containing azido groups only exhibited weak background fluorescence due to the intrinsic blue autofluorescence and nonspecific binding of the reagents (Figure 4A).

The azido-functionalized degummed silk fiber was reacted with excess amounts of DBCO-PEG4-DBCO to convert azido groups into DBCO groups. The DBCO-functionalized fiber was then reacted with a blue fluorescent reagent. The distinct blue fluorescence was observed on silk fiber (Figure 4B).

The above results demonstrated that the azido-functionalized silk can be readily modified with fluorophores in its native fibrous form by SPAAC, in this case yielding differently-colored fluorescent silk fiber. By changing fluorophores to other functional molecules, such as pigments, drugs, polymers, peptides, and proteins, silk fiber with any desired functions can be easily produced.

Silk can also be processed into various forms other than fibers, including aqueous solutions, hydrogels, transparent films, porous sponges, nanofibers, and nanoparticles [16,17,18]. We previously prepared a transparent film and porous sponge from the azido-functionalized silk and demonstrated that these silk materials were readily modified by SPAAC [9]. Since SPAAC requires no toxic catalysts or ligands and exhibits high chemical selectivity, variously-modified silk fiber or silk-based materials would be useful for a broad range of applications, including applications in the textile, healthcare, and medical fields.

## 3. Materials and Methods

### 3.1. Materials and Animals

All chemicals used in this study were of reagent grade and used as received. Cellulose powder was from Nacalai Tesque (Kyoto, Japan). 4-Azido-L-phenylalanine (AzPhe) was from Watanabe Chemical Industries (Hiroshima, Japan). Carboxyrhodamine 110 DBCO, sulforhodamine B DBCO, and DBCO-PEG4-DBCO were from Click Chemistry Tools (Scottsdale, AZ, USA). 3-Azido-7-hydroxycoumarin was from Baseclick (Neuried, Germany). A wildtype *B. mori* strain, Nichi509 × Nichi510, and a transgenic *B. mori* line, H06, which expresses the F432V mutant of BmPheRS in PSG [10], were used in this study. *B. mori* larvae were reared on an artificial diet, SilkMate PS (Nosan Corporation, Yokohama, Japan), at 22–26 °C.

### 3.2. Generation of the F_1_ Hybrid

Adult moths of the H06 line and Nichi509 × Nichi510 were mated to each other and their eggs were collected. To prevent eggs from entering diapause, the eggs were soaked in a dilute HCl (1.08 g/mL) at 46 °C for 2 min at around 20 h after oviposition [19]. Otherwise, at around 48 h after oviposition, the eggs were chilled at 5 °C for more than 2 months and then soaked in a dilute HCl solution (1.08 g/mL) at 48 °C for 3 min to break diapause. The HCl-treated eggs were washed with water, dried, and incubated at 25 °C for hatching.

### 3.3. Diet Preparation

AzPhe was mixed with SilkMate PM (dried form) (Nosan Corporation) at the desired ratio. Cellulose powder was added to normalize the dry weight among different conditions. Deionized water of 2.6 units of volume per unit weight of dry diet was added and heated for 1 h at 50 °C with gentle shaking to make uniform slurries. The slurries were heated for 5–10 min at 95 °C in an autoclave and stored in a refrigerator until use.

### 3.4. AzPhe-Incorporation Assay

At the third day of fifth instar, groups of three male larvae with similar average body weights were selected for each experimental condition. The AzPhe-mixed diet (0, 0.02, 0.05, or 0.1 wt% in dry diet) was administered once a day to three male larvae from the third day of fifth instar. When the average body weights of three larvae stopped increasing, each larva was individually transferred into a handmade paper box to allow it to start cocooning (at the seventh day in most cases). After 3–4 days, cocoons were separated from larvae or pupae and stored at −20 °C.

### 3.5. Urea Degumming

The amounts of fibroin in cocoons were calculated by estimating the proportion of the coating protein sericin by a urea degumming method as reported previously [7]. Briefly, aliquots of cocoons were heated in a degumming solution (8 M urea, 40 mM Tris-SO_4_ (pH 7)) at 80 °C for 10 min followed by washing with deionized water and then dried. The weights of fibroin in the cocoons were calculated from the weight loss by urea degumming.

### 3.6. In-Gel Digestion and Mass Analysis

Small pieces of cocoons were dissolved in 8 M LiBr solution at 35 °C at a concentration of 50 μg/μL. The dissolved solutions were used for in-gel digestion of FibL followed by MALDI-TOF-MS analysis as previously reported [14]. Briefly, the LiBr solution was subjected to SDS-PAGE and the FibL band was cut out from the gel. The gel pieces were destained, washed, and digested by Trypsin Gold (Promega, Fitchburg, WI, USA). The digested peptide fragments were extracted from the gel, dried, and subjected to MALDI-TOF-MS analysis using an autoflex III mass spectrometer (Bruker Daltonics, Billerica, MA, USA).

### 3.7. Production of Cocoons for the Reeling Experiment

At the third day of fifth instar, two groups of eighty larvae (including both male and female larvae) with similar average body weights were selected. The AzPhe-mixed diet (0 or 0.05 wt% in dry diet) was administered once a day to eighty larvae. When the average body weights of the larvae stopped increasing, each larva was individually transferred into a handmade paper box to allow it to start cocooning (at the seventh day in most cases). After eight days, cocoons were harvested, dried by heating up to 115 °C followed by gradual cooling for 6 h to 60 °C, and stored at room temperature until use.

### 3.8. Characterization of Raw Silk Fiber

Cocoons were cooked for circa 10 min using a VP-type vacuum cocoon cooking machine (HARADA, Okaya, Japan). Eight to nine cooked cocoons on average were reeled together to prepare raw silk of circa 27 D fiber size using a CT-2 type automatic reeling machine (NISSAN, Tokyo, Japan) at a reeling speed of 150 m/min at 40 °C.

To determine the degumming ratio, the sericin coating of the raw silk fiber was removed in 0.5% Na_2_CO_3_ at 95 °C for 20 min with gentle stirring. The fiber was then washed with water and dried.

The degumming ratio was calculated with the following equation:

$$\mathrm{Degumming}\;\mathrm{ratio}\;\left(\%\right)=\left(1-\frac{dry\;weight\;of\;degummed\;fiber}{dry\;weight\;of\;original\;fiber}\right)\times 100$$

The raw silk fiber was equilibrated at 20 °C and 65% RH for over 24 h. Tensile tests were performed with a gauge length of 100 mm and a strain rate of 150 mm/min using an RTG-1210 tensile testing machine (A&D, Tokyo, Japan) at 20 °C and 65% RH. Data collection and analysis were performed with TACT software (A&D).

### 3.9. Click Modification

The raw silk fiber was twisted at a rate of 685 turns/m using a TF-type twisting machine (TSUDAKOMA, Kanazawa, Japan) followed by heating in an autoclave for 10 min at a thermal setting at 110 °C. After twisting, the raw silk fiber was degummed in 0.5% Na_2_CO_3_ at 95 °C for 1 h with gentle stirring, washed with water, and dried. The degummed silk fiber was cut into circa 5 m lengths and wound on a piece of melamine foam. The fiber was put into a 15 mL centrifuge tube. The reaction mixture (5 μM carboxyrhodamine B DBCO or sulforhodamine B DBCO, 50 μM Tris-HCl (pH 8), 50% (*v*/*v*) DMSO) was poured into the tube. The tube was incubated overnight at room temperature with gentle shaking. The fiber was thoroughly washed with DMSO at 50 °C and deionized water at room temperature, and then dried.

To convert azido groups in silk fibroin into DBCO groups, the degummed silk fiber was first immersed in the reaction mixture (50 μM DBCO-PEG4-DBCO, 50 μM Tris-HCl (pH 8)) and incubated overnight at 37 °C with gentle shaking. The fiber was thoroughly washed with DMSO at room temperature and deionized water at room temperature, and then dried. The fiber was immersed in the reaction mixture (5 μM 3-azido-7-hydroxycoumarin, 50 μM Tris-HCl (pH 8), 50% (*v*/*v*) DMSO) and incubated overnight at room temperature. The fiber was thoroughly washed with DMSO at 50°C and deionized water at room temperature, and then dried.

Fluorescence on the fiber was observed with 254 nm UV light using a TL-2000 ultraviolet translinker (UVP; Upland, CA, USA).

## 4. Conclusions

An azido-bearing synthetic amino acid, AzPhe, can be incorporated into silk fiber during protein synthesis by expressing a mutant of BmPheRS in the PSGs of transgenic silkworms. The azido-functionalized silk fiber can be easily modified by click chemistry reactions. The highest efficiency of AzPhe incorporation was previously observed with the H06 transgenic line, which expresses the F432V mutant of BmPheRS. For industrial applications, it was necessary to improve the productivity of the azido-functionalized silk fiber. We generated an F_1_ hybrid of the H06 line with a high silk-producing strain and investigated its fibroin production and AzPhe incorporation into silk fiber. We demonstrated that fibroin production increased approximately 1.5 times by hybridization, whereas AzPhe incorporation was not affected. The mechanical properties of the azido-functionalized silk fiber exhibited no significant differences from normal fiber. Scaled-up production was performed using approximately 1000 transgenic silkworms, which produced circa 160 g of the azido-functionalized silk fiber. As a demonstration of click modification, we prepared differently-colored fluorescent silk fiber by SPAAC with fluorescent molecules. This study realized a rare opportunity to apply genetic code expansion methodology to the production of protein-based functional materials on an industrial scale.

## Figures and Tables

**Table 1:** Characterization of raw silk fiber reeled from the cocoons of the F_1_ hybrid, H06 × (Nichi509 × Nichi510), reared on a normal or an AzPhe-containing diet. Eighty larvae, including both male and female, were used for each experiment.

AzPhe (wt%)	Fiber Length ^1^ (m)	Raw Silk Ratio ^2^ (%)	Fiber Size ^3^ (D)	Degumming Ratio ^4^ (%)
0	714 ± 146	14.8 ± 1.6	3.73 ± 0.25	25.6 ± 0.3
0.05	631 ± 81	11.9 ± 0.1	3.55 ± 0.26	24.3 ± 8.7
	N.S.	*p* < 0.05	N.S.	N.S.

^1^ Length of raw silk fiber reeled out from one cocoon. ^2^ Ratio of raw silk fiber in a whole cocoon including a pupa. ^3^ Size of raw silk fiber shown in denier (D), with denier defined as the weight (g) of a 9000 m length of fiber. ^4^ Ratio of sericin coating in raw silk fiber removed by degumming (see Section 3.8). All values in this table are the average of three independent experiments shown with standard deviations (*n* = 3). Statistical significance by *t*-test is shown in the bottom column.

**Table 2:** Mechanical properties of raw silk fiber reeled from the cocoons of the F_1_ hybrid, H06 × (Nichi509 × Nichi510), reared on a normal or an AzPhe-containing diet. All values in this table are the average of three independent experiments with standard deviations (*n* = 3). In each experiment, 50 threads were tested and their results were averaged. Statistical significance by t-test is shown in the bottom column.

AzPhe (wt%)	Young’s Modulus (GPa)	Maximum Stress (MPa)	Maximum Strain (%)
0	14.1 ± 0.2	455 ± 20	20.5 ± 0.8
0.05	14.2 ± 0.5	454 ± 33	21.2 ± 1.2
	N.S.	N.S.	N.S.

## Supplementary Materials

Supplementary materials can be found at http://www.mdpi.com/1422-0067/20/3/616/s1.

## Abbreviations

AzPhe	4-azido-L-phenylalanine
DBCO	dibenzocyclooctyne
FibH	fibroin heavy chain
FibL	fibroin light chain
MALDI-TOF-MS	matrix-assisted laser desorption/ionization time-of-flight mass spectrometry
PEG	polyethylene glycol
PheRS	phenylalanyl-tRNA synthetase
PSG	posterior silk gland
SPAAC	strain-promoted azido-alkyne cycloaddition
