# Supplementary material for: Characterization and Scaled-Up Production of Azido-Functionalized Silk Fiber Produced by Transgenic Silkworms with an Expanded Genetic Code
Source: Int J Mol Sci. 2019 Jan 31;20(3):616. doi: 10.3390/ijms20030616 (PMC6387213; doi:10.3390/ijms20030616)
Supplement: Supplementary file 1 [file ijms-20-00616-s001.pdf]

-Supporting Information-

Characterization and Scaled-Up Production of Azido-Functionalized Silk Fiber Produced by Transgenic Silkworms with an Expanded Genetic Code

Hidetoshi Teramoto *, Masatoshi Iga, Hiromi Tsuboi, and Kenichi Nakajima

Division of Biotechnology, Institute of Agrobiological Sciences, National Agriculture and Food Research Organization (NARO)

* Correspondence: teramoto@affrc.go.jp; Tel.: +81-29-838-6162

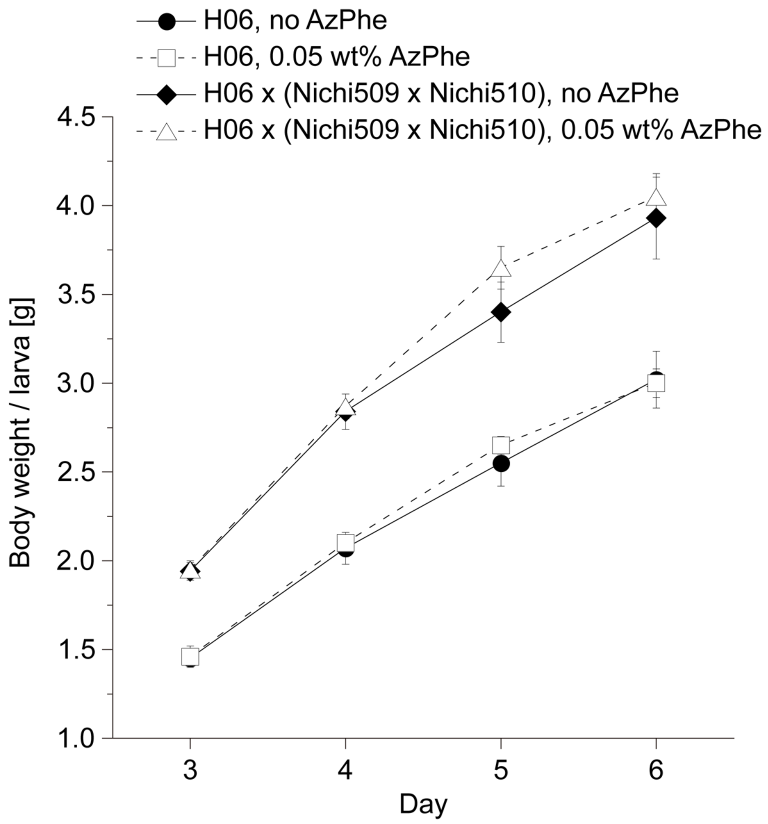

Figure S1. Larval growth of H06 and its F_1 hybrid, H06 × (Nichi509 × Nichi510), from their 3rd day to 6th day of 5th instar. Larvae were fed an artificial diet containing AzPhe (0 or 0.05 wt% in dry diet) from their 3rd day until the start of spinning. Larvae usually start spinning on the 7th day. All values in this figure are the average of four independent experiments, where each experiment employed three male larvae. The error bars represent standard deviations (n = 4).

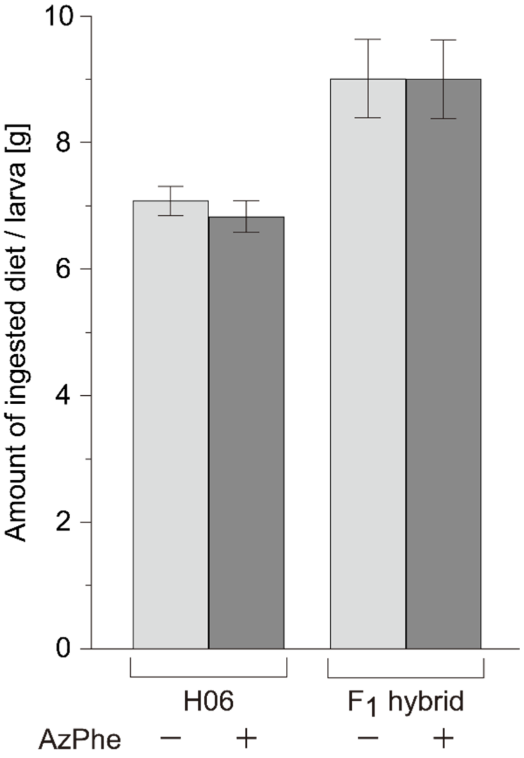

Figure S2. Diet ingestion of H06 and its F_1 hybrid, H06 × (Nichi509 × Nichi510). Larvae were fed an artificial diet containing AzPhe (0 or 0.05 wt% in dry diet) from their 3rd day until the start of spinning. All values in this figure are the average of four independent experiments, where each experiment employed three male larvae. The error bars represent standard deviations (n = 4).

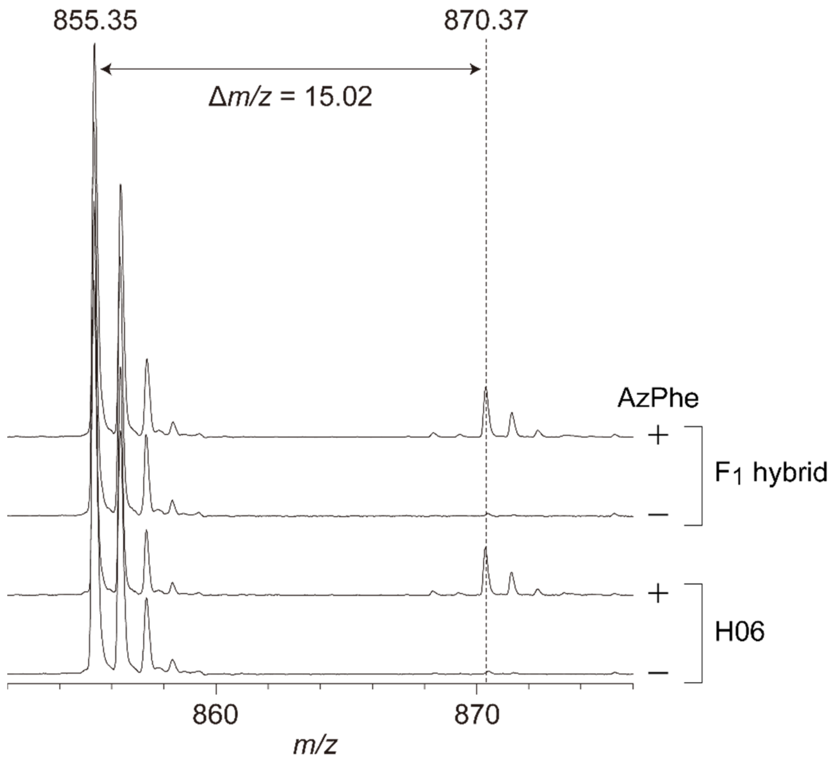

Figure S3. Representative MALDI-TOF-MS spectra of the Phe-containing peptide fragment derived from FibL (SGNFAGFR, $[M + H]^+$ = 855.41 Da). The parental peaks at circa 855 Da were standardized in all spectra. Replacement of one Phe to AzPhe gave a new peak at circa 870 Da, because the azido group in AzPhe was reduced to an amino group during the analytical procedure. The ratio of peak intensities, 870 Da / 855 Da, was used to estimate the AzPhe-incorporation rate in silk fibroin.
